# Validity of a Smartphone-Based Application for Determining Sprinting Performance

**DOI:** 10.1155/2016/7476820

**Published:** 2016-07-21

**Authors:** Robert Stanton, Melanie Hayman, Nyree Humphris, Hanna Borgelt, Jordan Fox, Luke Del Vecchio, Brendan Humphries

**Affiliations:** School of Medical and Applied Sciences, Central Queensland University, Bruce Highway, Rockhampton, QLD 4702, Australia

## Abstract

Recent innovations in smartphone technology have led to the development of a number of applications for the valid and reliable measurement of physical performance. Smartphone applications offer a number of advantages over laboratory based testing including cost, portability, and absence of postprocessing. However, smartphone applications for the measurement of running speed have not yet been validated. In the present study, the iOS smartphone application,* SpeedClock*, was compared to conventional timing lights during flying 10 m sprints in recreationally active women. Independent samples *t*-test showed no statistically significant difference between* SpeedClock* and timing lights (*t*(190) = 1.83, *p* = 0.07), while intraclass correlations showed excellent agreement between* SpeedClock* and timing lights (ICC (2,1) = 0.93, *p* = 0.00, 95% CI 0.64–0.97). Bland-Altman plots showed a small systematic bias (mean difference = 0.13 seconds) with* SpeedClock* giving slightly lower values compared to the timing lights. Our findings suggest* SpeedClock* for iOS devices is a low-cost, valid tool for the assessment of mean flying 10 m sprint velocity in recreationally active females. Systematic bias should be considered when interpreting the results from* SpeedClock*.

## 1. Introduction

The use of emerging technologies such as smartphones and tablets offers researchers and coaches opportunities to undertake physical performance assessments in the field, rather than the sports science laboratory. Current smartphone technology includes advanced computing capacity, inertial sensors, a global positioning system, and high speed video capacity [1]. Recently developed smartphone applications (APPs) for sport have capitalised on these advances and are shown to be valid and reliable tools to assess lower limb functional performance during countermovement jump [2, 3], drop jump [3], balance [4, 5], and maximal strength [6] tests. However, while the assessments of jumping performance and muscular strength are important characteristics of human performance, sprint speed is critical for success in a range of activities including football [7] and netball [8]. Yet unlike other physical performance characteristics, the assessment of sprinting capacity has received relatively less attention in the peer-reviewed literature [9].

Although fully automated timing systems using pressure sensitive starting blocks or photo finish recording systems remain the gold standard for the assessment of sprint running performance, such systems are rarely found outside athletic venues. They lack portability and are cost prohibitive to many teams, coaches, and athletes. Dual beam photocell systems are increasingly used due to their lower cost, portability, and capability to wirelessly send data to a handheld receiver. Dual beam systems exhibit greater accuracy compared to single-beam systems and have been recommended over single-beam systems [10]. Recent developments in error detection algorithms to address some of the shortcomings associated with single-beam systems have shown promising results. For example, the SmartSpeed (Fusion Sport, Coopers Plains, Australia), single-beam system with error detection, has been shown to comply with Australia's National Sport Science Quality Assurance standards, which require sprint testing systems to achieve a maximal typical error of 0.05 seconds over 30 m. D'Auria and colleagues [11] showed that the SmartSpeed system achieved a typical error of ≤0.03 seconds at distances of 5, 10, and 20 m.

Studies also show that video analysis of running performance has been shown to closely match fully automated timing systems [12] and dual beam photocell systems [13], even when recorded at 50 or 100 Hz. Therefore, even low-speed video capture may represent a low-cost and portable option to assess running performance. Recent innovations in smartphone technology have seen video capture speeds up to 240 Hz on iPhone 6. Coupled with improved image detection algorithms and computing power, smartphone APPs have been developed to allow coaches and researchers to determine running speed using these popular and low-cost tools. One such APP is* SpeedClock* (http://appmaker.se/?m=5&s=0).* SpeedClock* uses the iOS device camera to detect and record motion and, with the user input of a reference distance, calculates speed.* SpeedClock* can be used on a single standalone device, with the reference frame defined by the edges of the image detection field, or connected via Bluetooth with a second iOS device to record time over longer distances.

Although APPs such as* SpeedClock* are purported to accurately record sprint performance, to the best of our knowledge, there are currently no studies which demonstrate the validity of* SpeedClock*. Such studies are important in confirming the usefulness of emerging technologies and are of great interest to athletes, coaches, and researchers. Therefore, the purpose of the present study is to examine the validity of the* SpeedClock* APP using timing lights as a reference.

## 2. Methods

### 2.1. Participants

Participants were a convenience sample of 24 recreationally active females (>18 years) recruited via personal discussion with investigators. An information sheet outlining the purpose, risks, and benefits of participation was provided to all potential participants. Prior to inclusion in the study, participants were screened for musculoskeletal, neurological, or cardiorespiratory concerns which would contraindicate the performance of maximal sprinting, using stage one of the Adult Preexercise Screening System [14]. Written informed consent was obtained from all participants prior to the commencement of data collection. This study was approved by the institutional human research ethics committee.

### 2.2. Protocols

Participants stature was measured using a Seca 213 portable stadiometer (Seca GMBH, Hamburg) and weight determined using a Seca robusta 813 portable scale (Seca GMBH, Hamburg). All measures were performed according to standardised protocols [15]. All participants underwent a standardised warm-up comprising five minutes of light jogging, static and dynamic stretching, and a series of submaximal sprints. Participants then performed four maximal effort 20 m sprints, separated by five minutes of passive rest. Participants started on a line between the first set of timing lights and commenced the sprint in their own time. Standardised instructions were provided regarding maximal effort and to decelerate only when past the final set of timing lights. All sprints were performed in an indoor sports stadium (~26°C, 50% RH) on a suspended timber floor. Participants wore their usual running shoes and apparel.

### 2.3. Instruments

**Figure 1:**
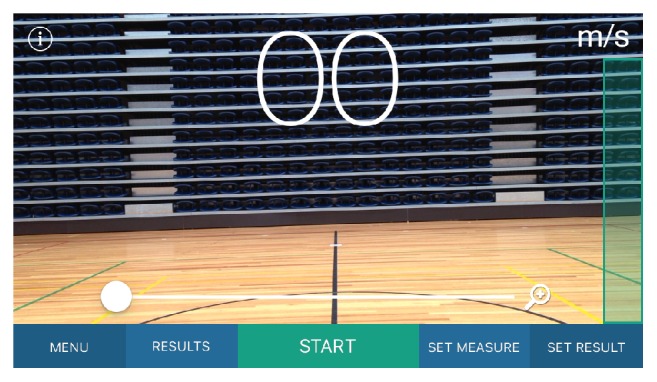
Screenshot of iPhone 5c showing motion detection zones at each edge of the field of view.

**Figure 2:**
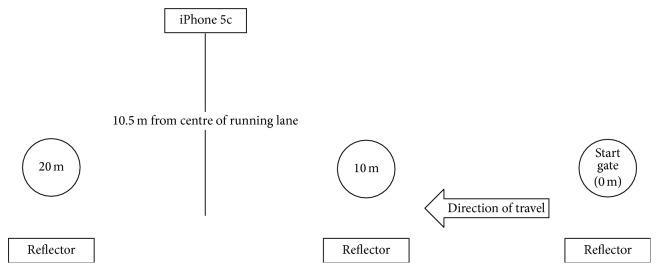
Positioning of the timing lights and iOS device.

Sprint times were recorded using SmartSpeed Pro timing lights (Fusion Sport, Coopers Plains, Australia), with gates at zero, 10, and 20 metres. This system uses a single-beam design to improve battery life and ease of setup, however, incorporates novel error detection algorithms to reduce false triggers. In the event of multiple triggers, the algorithm interprets the longest trigger as the true start time. Gates were set at a height of 1.0 m from the floor. Sprint times were converted to mean sprint velocity (m/s) for 0–10 m, 10–20 m, and 0–20 m using a standard linear motion equation (*v* = *d*/*t*). To examine the validity of the* SpeedClock* iOS application, the APP was installed on an iPhone 5c running iOS Version 9.2.1 (Apple Corporation, Cupertino, CA), which records video at 60 frames per second. In SpeedM (Motion) mode, the APP incorporates motion detection zones at each edge of the image which trigger and terminate data collection (Figure 1). For the present study, to ensure repeatability of the method and to minimise movement of the device, iPhone was mounted in a plastic tripod mount on a Velbon EX 330 tripod (Velbon Corporation, Tokyo, Japan), with the camera lens 1.0 m from the ground. The tripod was positioned 10.5 m perpendicular to the midpoint between the timing lights at 10 and 20 m, such that the motion detection zones of the application were aligned with the 10 and 20 m timing lights. The tracking sensitivity on the APP was set to 0.92 (arbitrary units) to avoid false triggers from background movement. In this manner, as the participant ran through the timing lights at 10 m, motion detection triggered data acquisition. Similarly, as the participant ran through the timing lights at 20 m, motion detection terminated data acquisition.  Figure 2 shows the positioning of the timing lights and iOS device. Mean velocity (m/s) for the flying 10 m sprint was displayed on-screen and manually recorded for later analysis.

### 2.4. Statistical Analysis

Descriptive statistics were calculated for participant data. All four flying 10 m sprints recorded simultaneously using timing lights and the* SpeedClock* application were used for analysis. Independent sample *t*-tests (two-tailed) were used to identify differences in average velocity obtained from the timing lights and* SpeedClock* APP. Between-device agreement was examined using intraclass correlation coefficients (ICC 2,1) with 95% confidence intervals (95% CI) and interpreted according to Munro [16]. Bland-Altman plots were then constructed using Microsoft Excel (Microsoft Corporation, Redmond, USA) to visualise the level of agreement between average velocity obtained from the timing lights and the* SpeedClock* APP. With the exception of Bland-Altman plots, all statistical analyses were performed using Statistical Package for the Social Sciences (SPSS), Version 22 (IBM Corporation, Chicago, Ill). Statistical significance was accepted at an alpha level of *p* < 0.05.

## 3. Results

**Figure 3:**
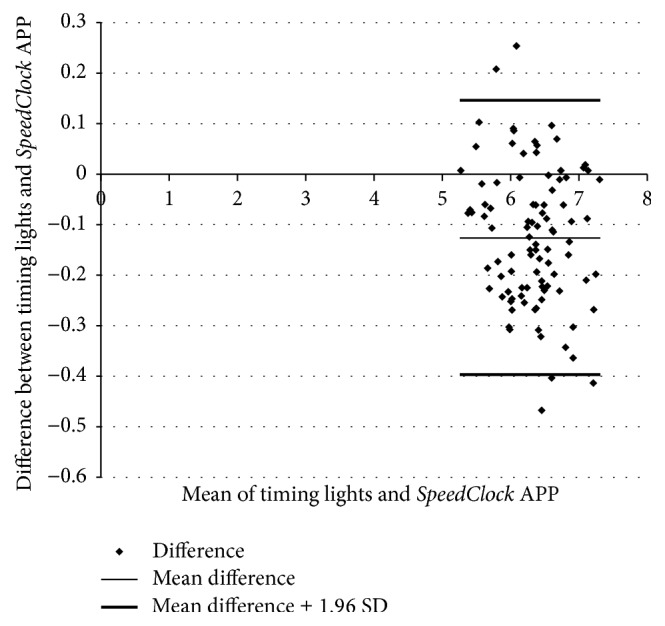
Bland-Altman plot depicting the level of agreement between timing lights and* SpeedClock* application for 10 m sprinting.

Twenty-four recreationally active females (mean age 26.6 ± 5.4 years, mean body mass index 25.0 ± 3.1 kg·m^−2^) provided informed consent and participated in the study. No adverse events were reported. Results for mean flying 10 m sprint times are shown in Table 1. Independent samples *t*-test showed no statistically significant difference in mean flying 10 m sprint velocity between data from the timing lights (6.47 ± 0.49 sec) and the* SpeedClock* APP (6.31 ± 0.48 sec) (*t*(190) = 1.83, *p* = 0.07). Intraclass correlations (ICC 2,1) showed excellent agreement in mean flying 10 m sprint velocity between data from the timing lights and the* SpeedClock* APP (ICC (2,1) = 0.93, *p* = 0.00, 95% CI 0.64–0.97). Bland-Altman plots to visualise the difference between mean flying 10 m sprint velocity determined by timing lights and* SpeedClock* APP are shown in Figure 3. A small systematic bias (mean difference = 0.13 seconds) shows the APP consistently gave slightly lower values compared to the timing lights.

## 4. Discussion

To the best of our knowledge, no other studies comparing APP-based measures of sprint running speed have been published in the peer-reviewed literature. As such this study makes an important contribution to the exercise and sports science domain. The findings from the present study suggest that the* SpeedClock* APP installed on an iPhone 5c recording video at 60 frames per second is a valid measure of average flying 10 m sprint velocity in recreationally active females.

A number of other studies have examined the use of video technology in determining sprint running performance. For example, Haugen and colleagues [12] reported no systematic variation between Brower dual beam infrared timing and Dartfish-based video analysis of 40 m sprint times in national level male and female track athletes. In another study, Harrison and colleagues [13] reported the validity of video recorded at 50 Hz and at 100 Hz, to determine mean sprint velocity over 3 m. No statistically significant differences and excellent ICCs were observed between a laser sports measurement system and video recorded at either frame rate. However, at medium to fast sprinting velocities, video recorded at 100 Hz compared more favorably with laser-derived measures than video recorded at 50 Hz, due to the higher sampling rate. In the present study, mean flying 10 m sprit velocity recorded using the* SpeedClock* APP was not significantly different to that recorded using dual beam timing lights. Moreover, ICCs showed excellent agreement with Bland-Altman plots indicating only a small degree of systematic bias, with a mean difference of −0.13 seconds (1.97%). Although this level of mean difference may be significant with respect to changes in sprint performance over time, these values are likely within expected test-retest reliability of sprinting performance, and researchers should examine the smallest worthwhile change in performance to determine if a real change has occurred.

One notable difference between the timing lights and the* SpeedClock* APP is that the timing lights report data to three decimal places, while the* SpeedClock* APP reports data to two decimal places. Therefore, we reran our ICC analysis using velocities based on timing light data under similar conditions, firstly truncated and then rounded to two decimal places. The resultant analyses were not substantially different from our initial findings (ICC (2,1) = 0.92, *p* = 0.00, 95% CI 0.68–0.97, and 0.93, *p* = 0.00, 95% CI 0.64–0.97, resp.) and do not change our interpretation of the validity of this APP.

Emerging technologies such as the use of smartphone applications in sport have the potential to provide athletes, coaches, and researchers with additional data not otherwise available with a single laboratory based system. For example, when using* SpeedClock*, an image of the participant can be captured midway through the image capture field. This may provide valuable data to assess running technique, and, since the image is stamped with the mean velocity, it serves as a permanent record of the attempt. Unlike GPS-based system,* SpeedClock* is video-based and therefore can be used indoors. Finally, unlike manual stopwatch timing, the data is not affected by parallax error when standing at the start or finish line, and in the case of* SpeedClock*, two devices can be used together in a manner not unlike timing gates. In general, smartphone-based APPs for sprint performance assessment offer low-cost, portability, and ease of use, not otherwise available with laboratory based systems, and their widespread applicability to training, rehabilitation, and research warrants further investigation.

A strength of the present study is the use of Bland-Altman plots to examine systematic bias between methods used to determine sprint velocity. However, a potential limitation is the use of the iPhone 5c, which records video at 60 frames per second. Future studies should examine the use of high speed video on newer iOS devices as the increased resolution afforded by the higher frame rate may improve accuracy and reduce systematic bias. Future studies should also examine other speed recording APPs on Android operating systems, since, at the present time,* SpeedClock* is only available on iOS and, although iOS devices are widely utilised, they are not the sole software platform. Finally, future studies should examine the validity of the* SpeedClock* or similar APPs over shorter and longer distances and in clinical populations.

## 5. Conclusions

*SpeedClock* for iOS devices is a low-cost, valid tool for the assessment of mean flying 10 m sprint velocity in recreationally active females. Further studies are required in different populations and settings to generalise the findings from the present study.

## Figures and Tables

**Table 1:** Mean flying 10 m sprint times for timing lights and *SpeedClock* APP.

Timing lights	*SpeedClock* APP
6.48 ± 0.49	6.31 ± 0.48
